# Adapting blended learning for large undergraduate medical classes: insights from the 4C/ID model

**DOI:** 10.5116/ijme.67bb.0fa4

**Published:** 2025-03-14

**Authors:** Birgitte Schoenmakers, Pascal Ryckaert, Bert Bammens, Dirk Van Raemdonck, Vasiliki Andreou

**Affiliations:** 1 Academic Center for General Practice, Department of Public Health and Primacy Care, KU Leuven, Belgium; 2 Teaching Programs Coordination, Faculty of Medicine, KU Leuven, Belgium; 3 Department of Chronic Diseases and Metabolism, KU Leuven, Belgium

**Keywords:** Blended learning, Four-Component Instructional Design, undergraduate medical classes, quality of care, healthcare

## To the Editor

We would like to share the findings from a study conducted at KU Leuven, where we adapted the Four-Component Instructional Design (4C/ID) 1 model for large undergraduate medical student groups. This adaptation was applied to the course Quality of Care, which integrates the CanMEDS roles to foster a holistic understanding of healthcare. The course focuses on patient encounters, primary care, and quality of care, including aspects such as patient safety, healthcare models, and care organization.

The goal of our study was twofold: to adapt the 4C/ID model for large class groups without losing the core pedagogical principles and to examine how blended learning methods could improve the learning experience and manage the workload of educators. This adaptation aimed to create a more flexible learning environment that incorporates both online and traditional classroom interactions.

The 4C/ID model, as developed by van Merriënboer, provides a structured approach to complex learning, particularly suited for real-life professional tasks. In the context of medical education, this model is crucial for developing clinical reasoning and integrating knowledge from various healthcare domains.^1^ However, the challenge lies in adapting such a model, initially designed for small, interactive groups, to large class settings. At KU Leuven, the Quality of Care course is compulsory for third-year medical students and enrolls around 500 students per year.

Traditionally, this course was taught in smaller groups, but as student numbers grew, it became necessary to redesign the course delivery without compromising the quality of education. The course adaptation incorporated blended learning techniques, including interactive lectures, online exercises, guest speaker sessions, and workshops, all supported by the Blackboard Toledo Ultra platform.

We conducted a retrospective mixed-method study over three academic years (2017-2021) to evaluate the impact of the course modifications. Student satisfaction and the effectiveness of the blended learning approach were assessed using a validated university-wide survey. The survey included 14 items that covered themes such as teaching quality, study load, perceived learning outcomes, course communication, and the structure of the learning material.

The survey employed a Likert scale ranging from 1 to 6, with a score of 4 indicating satisfactory performance. Items scoring below 4 were considered areas needing improvement. In addition to the quantitative data, students provided qualitative feedback through open-ended questions about aspects of the course they liked and those that required modification. The survey response rate ranged from 30% to over 50% across the evaluation rounds.

Each evaluation round was followed by a formal review by a commission comprising faculty, educational staff, and student representatives. This commission discussed the results and made recommendations for course improvements based on student feedback. These recommendations were then used to adjust the course structure, learning objectives, and assessment methods.

In the first evaluation round (2017-2018), the results indicated several areas that required attention. Students rated the enthusiasm of the instructors highly, with a score of 4.41 out of 6, reflecting their appreciation for the instructors’ passion and engagement. However, many other areas fell below the threshold score of 4. Students expressed concerns about

the clarity of learning objectives (scoring 3.08), the logical structure of the course (scoring 3.42), and the representativeness of the exam (scoring 3.56). They also mentioned difficulties with communication regarding assessment methods and found the learning content too dense and sometimes irrelevant.

In response, significant revisions were made to the course before the second evaluation round (2019-2020). These included reorganizing the course materials, clarifying learning objectives, and enhancing communication about the structure and expectations of the course. The revised course also incorporated more peer interactions and practical exercises to help students link theoretical knowledge with real-life clinical practice. These changes resulted in improved scores in most areas, with teaching comprehensibility scoring 4.50, course cohesion scoring 3.84, and overall satisfaction rising to 3.95.

However, students continued to express concerns about the exam format, with the representativeness of the exam dropping to 2.39. This highlighted a disconnect between the course content and the assessment, suggesting that the exam did not accurately reflect the learning outcomes or adequately test the students’ understanding of key concepts.

By the final evaluation round (2020-2021), further refinements were made to both the course and the exam. These adjustments included providing sample exam questions, offering more detailed instructions on the learning objectives, and reducing the volume of learning materials. By this time, all but one survey item scored above the threshold of 4, with overall satisfaction rising to 4.66. However, learning objectives (3.74) and the exam format (4.02) remained challenging for students. Although improvements were made, students still found the learning objectives somewhat unclear and expressed frustration with the multiple-choice exam format, preferring more open-ended or oral assessments.

Adapting the 4C/ID model for large class groups presented unique challenges. One of the primary difficulties was maintaining student engagement and fostering deep learning in such a large cohort. While the blended learning approach, incorporating both in-person and online elements, was generally well-received, several issues persisted, particularly regarding the clarity of learning objectives and the structure of the exam.

The iterative evaluation process was crucial in identifying these issues and making necessary adjustments. For example, students’ feedback about the overwhelming amount of learning material led us to categorize resources into “must know,” “nice to know,” and “for the interested,” which helped students focus on essential content. Additionally, the introduction of sample questions and better communication about the exam format addressed some concerns, though the multiple-choice format continued to be a pain point for many students.

From a pedagogical standpoint, the blended learning approach provided flexibility and personalized learning opportunities. Students appreciated the interactive lectures, the involvement of guest speakers, and the practical relevance of the course content. The workshops, in particular, allowed students to apply theoretical knowledge in a controlled environment, helping them build clinical reasoning skills. However, these benefits were sometimes overshadowed by the lack of clear learning objectives and the exam format, which students felt did not align with the course content.

Our experience with adapting the 4C/ID model for large class groups in undergraduate medical education highlights the importance of continuous evaluation and adaptation. Blended learning offers many advantages, including flexibility and personalized learning paths, but it also requires careful design and ongoing refinement to meet the needs of students and educators alike.

The findings from this study emphasize the need for clear learning objectives, well-aligned assessments, and effective communication between educators and students. While we made significant strides in improving the course structure and content delivery, the exam format remains an area for further development. In large classroom environments, multiple choice questions are the most practical assessment method; however, they often lack the ability to capture deeper cognitive insights. Implementing open-ended questions or utilizing more advanced adaptive questioning formats could potentially address this limitation.

In conclusion, the adaptation of the 4C/ID model for large class groups has proven feasible, but it requires a collaborative approach and a willingness to adjust based on student feedback. This process of continuous improvement is essential for ensuring high-quality education and preparing future medical professionals for the complexities of healthcare practice.

### Conflicts of Interest

The authors declare that they have no conflict of interest.
